# Errata

**Published:** 1892-10

**Authors:** 


ERRATA.—In our last issue, page 108, in the article on cholera, there was an accidental omission of a line which caused a ridiculous mistake. Speaking of the *standard solution* for disinfecting feces, we said "corrosive sublimate 4 oz., sulphate copper 1 lb., to a gallon of water; of this, eight ounces should be used for each stool." It should have read, "*Add this solution to ten gallons of water*, and of this solution use eight ounces to each stool." Quite a difference in strength.

Our remarks with regard to looseness of the bowels have been criticised. The idea sought to be conveyed was that any looseness of the bowels, in the absence of the specific microbe of cholera, could never run into cholera. Cholera is frequently preceded by a looseness, but a looseness is not necessarily a forerunner of cholera. A cholera patient may die without any action of the bowels at all, yet it is the exception and not the rule.

In another article, on page 106, the compositor made us spell "presumptious" "presumptuous," and the proof reader failed to catch on. Oh, there are troubles and trials even in an editor's existence, as rosy-hued (?) and pleasant (?) as it generally is. Our only solace is the hope that those who know us will at least give us credit for knowing how to spell.

